# The role of immune cells and associated immunological factors in the immune response to spinal cord injury

**DOI:** 10.3389/fimmu.2022.1070540

**Published:** 2023-01-05

**Authors:** Huaguo Tang, Yuanjie Gu, Lei Jiang, Gang Zheng, Zhuoer Pan, Xiugui Jiang

**Affiliations:** ^1^ Department of Hand and Foot Surgery, Zhejiang Rongjun Hospital, Jiaxing, China; ^2^ Department of Neurosurgery, The Central Hospital Affiliated to Shaoxing University, Jiaxing, China; ^3^ Department of Orthopedics, Zhejiang Rongjun Hospital, Jiaxing, China

**Keywords:** spinal cord injury, immune response, immune cells, immune cytokines, therapeutic targets

## Abstract

Spinal cord injury (SCI) is a devastating neurological condition prevalent worldwide. Where the pathological mechanisms underlying SCI are concerned, we can distinguish between primary injury caused by initial mechanical damage and secondary injury characterized by a series of biological responses, such as vascular dysfunction, oxidative stress, neurotransmitter toxicity, lipid peroxidation, and immune-inflammatory response. Secondary injury causes further tissue loss and dysfunction, and the immune response appears to be the key molecular mechanism affecting injured tissue regeneration and functional recovery from SCI. Immune response after SCI involves the activation of different immune cells and the production of immunity-associated chemicals. With the development of new biological technologies, such as transcriptomics, the heterogeneity of immune cells and chemicals can be classified with greater precision. In this review, we focus on the current understanding of the heterogeneity of these immune components and the roles they play in SCI, including reactive astrogliosis and glial scar formation, neutrophil migration, macrophage transformation, resident microglia activation and proliferation, and the humoral immunity mediated by T and B cells. We also summarize findings from clinical trials of immunomodulatory therapies for SCI and briefly review promising therapeutic drugs currently being researched.

## 1 Introduction

Spinal cord injury (SCI) is a devastating neurological condition that most commonly results from vertebral fractures caused by traumatic accidents, including motor vehicle accidents, falls, and sports injuries. A few SCIs result from non-traumatic events such as infections or vascular damage (1). The clinical manifestations of SCI include loss of sensory and/or motor function below the level of injury. The symptoms can be partial or complete depending on the degree of injury and its location on the spinal cord (1). The estimated global incidence of total SCI in 2019 was 900,000, with an age-standardized incidence rate of 12 per 100,000 (2). Currently, patients who survive after severe SCI find it difficult to achieve complete recovery owing to the limited regenerative capacity of the lesioned spinal cord. As a result, SCI can not only causes serious disability but can also place great economic burden on patients’ families and society at large (3). Therefore, it is crucial to clarify the underlying cellular and molecular mechanisms of SCI pathophysiology and investigate novel therapeutic targets for intervention. After SCI onset, the primary injury caused by mechanical damage is the mechanical disruption of tissues and subsequent edema (4). This is followed by a secondary injury cascade that develops over the subsequent weeks and/or months. This involves vascular dysfunction (5), ischemia (6), excitotoxicity (7), ionic dysregulation (8), oxidative stress (9), neurotransmitter toxicity (10), lipid peroxidation (11), necrosis/apoptosis (12), immune-inflammatory response (13), and Wallerian degeneration and scar formation (14). These biological responses in secondary injury cause further damage to the spinal cord. Nevertheless, proper interventions in these processes can aid tissue regeneration and functional recovery after SCI.

The immune response appears to be the key molecular mechanism underlying these various pathophysiological processes and the main factor affecting the outcome of SCI (15). Immune response to SCI orchestrates the activation of different immune cells and the generation of various immunological factors. After SCI, astrocytes, the primary resident cells in the spinal cord, react rapidly by producing immunological mediators and recruiting or activating immune cells, including macrophages/monocytes, neutrophils, microglia, and T and B cells. The immunological mediators released by immune cells include not only inflammatory cytokines such as interleukin (IL)-1β, IL-6, and tumor necrosis factor (TNF)-α, but also chemicals such as chondroitin sulfate proteoglycans (CSPGs). With recent developments in biotechnology, a more precise method to understand the heterogeneity of immune cells and immunological factors is now available. This provides novel perspectives for investigating new therapeutic targets. In this review, we focus on the latest findings on (i) the pathophysiological mechanisms underlying SCI, (ii) heterogeneity of these immune components and their roles in SCI, and (iii) clinical trials and promising basic research on immunomodulatory drugs for SCI.

## 2 The role of the immune response in pathophysiological processes in spinal cord injury

In traumatic SCI, primary injury is predominantly caused by the compression of,or distraction of the spinal cord resulting from dislocated bone fragments, discs, and ligaments (16). In traumatic SCI, primary injury is primarily caused by the compression of the spinal cord (17). Primary injury leads sequentially to local hemorrhage, edema, ischemia, and hypoxia at the site of injury (16). The disruption of blood vessels at the site of SCI results in hemorrhage and increased permeability of the blood–brain barrier (BBB)/blood–spinal cord barrier (BSCB). Following the release of vasogenic and cytotoxic factors, active chemicals infiltrate to site of injury, which causes tissue edema. Subsequent blood flow reduction at the injury site causes ischemia and hypoxia in local neurons and glial cells. Early surgical decompression of the spinal cord within 24 h of SCI onset is the best treatment to reduce neuronal damage caused by primary injury (18). Biological responses in the primary injury are significant proponents of the development and progression of a pathophysiological cascade of secondary injury, including oxidative stress, ischemia, BSCB disruption, inflammatory and immune response, apoptosis, and mitochondrial dysfunction. In acute SCI, secondary injuries are usually categorized into four phases: the acute phase (<48 h), subacute phase (2–14 days), intermediate phase (14–180 days), and chronic phase (>180 days) (16, 19). In the acute phase, a disrupted BBB/BSCB results in astrocyte polarization, the infiltration of immune cells, including neutrophils, monocytes, and T cells, into the injured site of the spinal cord, and the proliferation of resident microglia (15). These cells release pro-inflammatory and chemotactic factors, triggering inflammation, lipid peroxidation, necrosis, edema, oxidative stress, calcium influx, ion disturbance, and excitotoxicity (13). In the subacute phase, the critical characteristics include the commencement of reactive astrogliosis at the injury site and infiltration of macrophage (20, 21). The resolution of edema and BSCB repair are also initiated in this phase (19). In the intermediate phase, reactive astrogliosis continues and glial scars start maturing, which is followed by axonal sprouting of the corticospinal tract and reticulospinal fibers (22). The chronic phase is typically characterized by the stabilization of scar formation, cysts/syrinx formation, and Wallerian degeneration (16).

**Figure 1:**
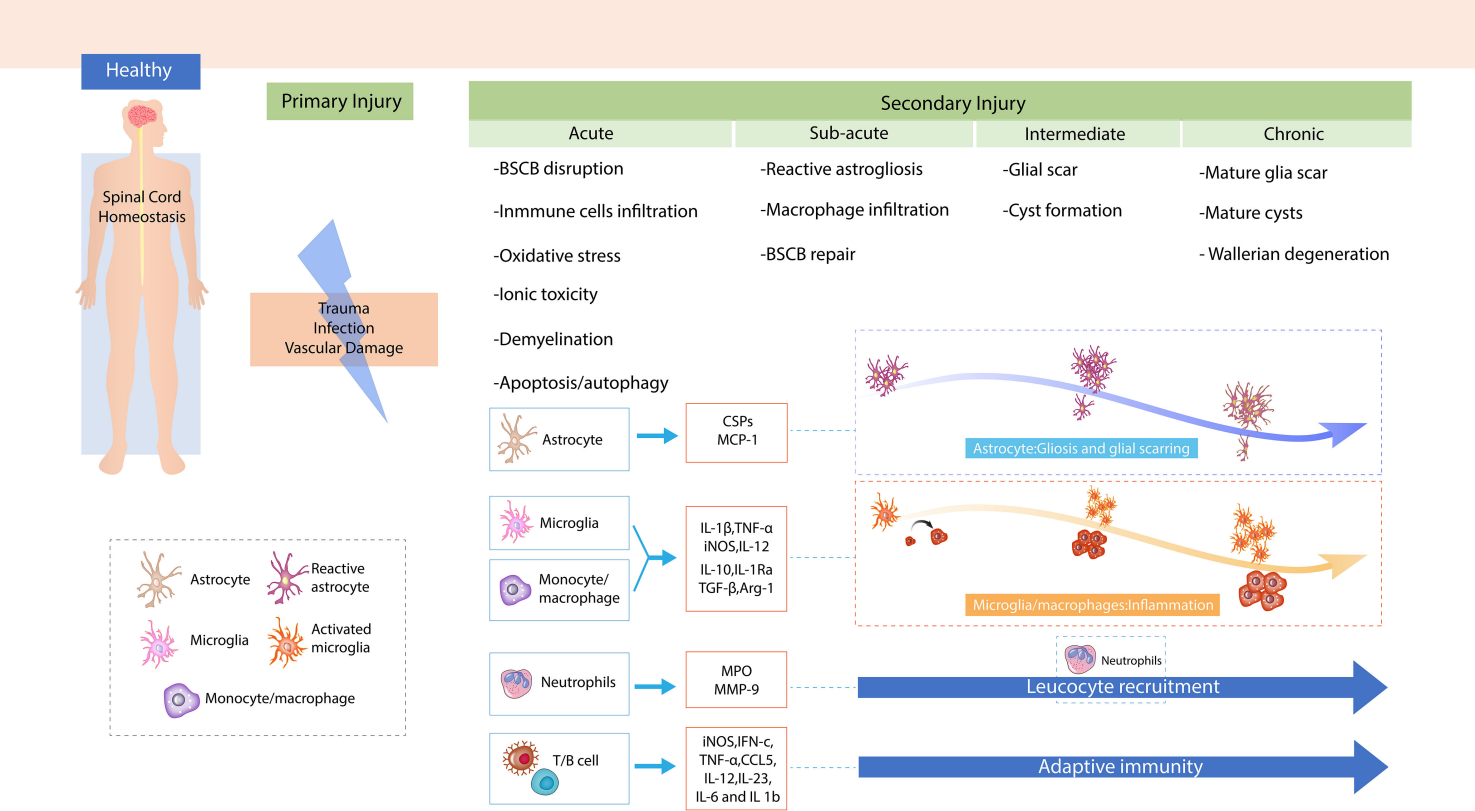
The mechanism of spinal cord injury (SCI). Demonstration of primary injury and secondary injury, including four phases of SCI after trauma to the spinal cord.

The immune response contributes to each phase of secondary injury and affects the clinical outcome. Moreover, immune cells and related immunological factors also modulate the pathophysiological reactions in SCI (**Figure 1**). Nguyen et al. reported that neutrophil infiltration led to cytotoxic tissue damage *via* the release of excessive cytokines, prostaglandins, and toxic granules, and also caused oxidative stress (23). Chen et al. demonstrated that a stronger local immune microenvironment inhibited apoptosis, thus aiding recovery in murine SCI (24). Mohrman et al. showed a strong immune response associated with the reduction of neuronal signal transmission and better recovery of the SCI animal model (25). Collectively, a better understanding of the immune response after SCI may help investigate effective therapeutic targets for protecting tissue and functional loss in response to SCI.

## 3 How are the functions of immune cells and immune factors orchestrated in the pathophysiology of spinal cord injury?

### 3.1 Astrocytes

#### 3.1.1 The dual roles of astrocytes in spinal cord injury pathophysiology

Astrocytes are the most abundant cells in the central nervous system (CNS) and contribute to the functional and structural homeostasis of the CNS (26) by regulating the balance of neurotransmitter ions (27) and maintenance of the BBB (28). Astrocytes do not belong to the immune system, but they play essential roles in immune responses to SCI by, for example, recruiting immune cells and secreting immunomodulatory molecules, such as transforming growth factor (TGF-β) and TNF-α. After SCI, astrocytes in the quiescent state (i.e., naïve astrocytes) gradually shift to an activated state (i.e., reactive astrocytes), forming a glial scar, which has dual roles that are either protective or detrimental to the recovery of injured tissues and neurological functions of patients (26).

Astrocytes proliferate and migrate in an overlapping manner to the lesion site, and are arranged into glial scars around the area of injury. With this, they form a network of tangles with other cells (including fibroblasts and oligodendrocyte precursor cells, among other cells) and secrete various cytokines to participate in the subsequent inflammation and repair process (29, 30). Glial scars prevent the spread of necrotic and apoptotic cells and restrict inflammation to the lesioned area (20). Simply eliminating astrocytes severely affects the pathology of and recovery after SCI, such as by causing more severe inflammation, increasing lesion volume, and worsening motor dysfunction. Although the physical and molecular properties of scars limit the spread of inflammation to the area of injury, the accumulation of excess astrocytes follows. The physical barrier also hinders the damage to axon regeneration and scaffolds disruption in the extracellular matrix, which will soften the tissue and lead to the failure of axon regeneration (31). In the final phase, astrocytes constitute the predominant cell type in the injury site (32) and form scar tissue as a barrier between necrotic and normal tissues (33). An *in vitro* study using primary astrocytes found that transforming growth factor β3 (TGFβ3) exerts neuroprotection effect and reverses the neurotoxic phenotype of astrocytes induced by aligned poly-L-lactic acid fibers (34). The formation of astrocyte scars aids rather than inhibits the regeneration of axons in the CNS. Blocking the formation of astrocytic scars significantly reduces the regrowth of laminin-dependent sensory axons and related molecules across scar-forming astrocytes (35). A robust stimulation was observed in the regeneration of propriospinal axons in the astrocyte scar border as well as in the lesion cores of non-neural tissue (35). The conditional deletion of sterile alpha and TIR motif-containing 1 (SARM1) in neurons and astrocytes improved the functional recovery of behavior performance post-SCI (36). After SCI, the traumatized spinal cord is protected by neuron-derived exosome-transmitted miR-124-3p *via* inhibited activation of neurotoxic microglia and astrocytes (37).

Traditional terms associated with astrocytes include reactive astrocytes and reactive astrogliosis. The modern concept of astrocyte polarization was proposed by Virchow, who described astrocyte polarization after trauma and neurodegenerative injury (38). In 2017, Liddelow et al. first proposed that reactive astrocytes can be characterized as inflammatory astrocyte 1 (A1) and neuroprotective astrocyte 2 (A2). The activation of A1-type astrocytes was induced by microglia in neuroinflammation, with C3 as a surface marker. Activated microglia *in vitro* and *in vivo* secrete IL-1α, TNFα, and C1q to activate A1 astrocytes (39). Based on this new classification method, recent studies have provided a new perspective. In 2021, Zhang et al. found that miR-21a-5p can aggravate the inflammatory response after traumatic SCI by increasing A1 polarization *via* the inhibition of the CNTF/STAT3/Nkrf signaling pathway (40). In the same year, in another study, Li et al. demonstrated that heat shock transcription factor 1 plays a key role in suppressing the excessive increase in neurotoxic A1 astrocytes (41). Wang et al. showed that the transformation of A1/A2 reactive astrocytes may be associated with functional recovery after SCI (42). Furthermore, a recent study based on single-cell RNA sequencing demonstrated that astrocytes in the injured tissue of SCI showed the altered expression of biomarkers, including Atp1b2, S100a4, Gpr84, C3/G0s2, GFAP/Tm4sf1, and Gss/Cryab, which can help achieve a more precise classification (43).

#### 3.1.2 Pivotal immune chemicals released by astrocytes

Following SCI, reactive astrocytes in the glial scar produce high levels of CSPGs (44), which were initially known to inhibit axonal growth and regeneration (45), but were later found to be beneficial in spinal cord recovery (46). CSPGs are extracellular matrix proteins that contribute to the interactions between the matrix and the immune system (47). CSPGs bind to chemokines/cytokines, growth factors, and pro-inflammatory cytokines required for immune cell growth and recruitment, thereby modulating the immune system (47). Astrocytes also secrete monocyte chemoattractant protein-1 (MCP-1), which is a chemokine inhibiting the expansion of the lesioned spinal cord tissue by attracting M1 macrophages (48). Other chemokines, such as chemokine C–C motif ligand 2 (CCL2) (49) and C–X–C motif ligand 1 (CXCL1) are produced by astrocytes. These chemokines will intensify the recruitment of neutrophils and pro-inflammatory macrophages after SCI (50). Moreover, astrocytes release anti-inflammatory cytokines, such as IL-10 and TGF-β, which can enhance the transformation of microglia/macrophages to a pro-regenerative M2-like phenotype (51, 52).

### 3.2 Neutrophils

#### 3.2.1 The dual roles of neutrophils in spinal cord injury pathophysiology

Neutrophils are fundamental members of the innate immune system (51). In humans, neutrophils are derived from bone marrow hematopoietic stem cells and are known as polymorphonuclear leukocytes. Although neutrophils have a short lifespan (less than 24 h), they have a generation rate of 1 × 10^11^ cells/day, which makes them the most abundant granulocytes in peripheral blood (53). In SCI, neutrophils are the first immune cells that arrive at the site of injury, i.e., within 3 h of SCI, and remain at the site for 3 days post injury. Large number of neutrophils gather at the site of injury within 24 h post SCI (54). The infiltration of neutrophils to the site of SCI exerts adverse effects *via* the secretion of neutrophilic tissue-damaging factors (54). These unfavorable factors trigger biological responses, such as oxidative stress, inflammation, pro-inflammatory cytokins release, and BSCB disruption, which interrupts vascular formation. In mice, neutrophil accumulation inhibition after SCI can improve white matter sparing and promotes rapid neurological recovery (55).

Although numerous studies have paid attention to the deleterious role and effect of neutrophils in SCI, we should take into account that neutrophils are heterogeneous, and the recruitment of neutrophil post SCI may not completely reflect the exacerbation of injury. Early pathogenesis of the contused spinal cord and long-term neurological recovery are mediated by neutrophils and monocytes (56). As the initial responders at the site of injury, neutrophils and monocytes can initiate the clearance of debris and generate pro-inflammatory cytokines that recruit other immune/inflammatory cells, such as macrophages, to engulf residual debris and promote tissue repair (57). Sas et al. revealed that a new subgroup of neutrophils (i.e., CD14^+^ Ly6G*^low^*) increases the survival of neurons and promotes axon regeneration in the CNS (58).

#### 3.2.2 Pivotal immune chemicals released by neutrophils

Neutrophils activate more immune cells for migration to the injury site and clear debris by secreting pro-inflammatory factors. Neutrophils were also shown to participate in inflammation and tissue healing *via* the release of secretory leukocyte proteases (59). Myeloperoxidase (MPO) is one of the most abundantly expressed proteins in neutrophils. It is a peroxidase enzyme present in the granules of neutrophils. The enzyme induces the formation of hypochlorous acid to eliminate the invasion of pathogens; at the same time, it can also cause unintentional host tissue injury. Release of MPO attracts the aggregation of neutrophils, contributing to sustained damage (60). Matrix metallopeptidase 9 (MMP-9), secreted by neutrophils in response to SCI (61), has been shown to be involved in the BBB degradation, facilitating the entry of cells, such as inflammatory cells, into the CNS (62). Members of the MMP family affect functional recovery after SCI by modulating the BSCB (63–65).

### 3.3 Microglia

#### 3.3.1 The roles of microglia in the pathophysiological process of spinal cord injury

Microglia are the resident immune cells of the CNS. Thus, they are the early immune cells that respond to tissue damage after SCI (66). Microglia account for approximately 10% of all CNS cells. These cells help maintain CNS homeostasis through continuous interactions with neuronal and non-neuronal cells. It is the first line of immune defense in the CNS. Microglia constantly remove damaged nerves, plaques, and infectious substances from the CNS. Activated microglia play an important role in neurodegenerative diseases such as Parkinson’s disease, multiple sclerosis, and Alzheimer’s disease (67). Microglia are activated soon after initial damage in the spinal cord. They mediate morphological change, neurotoxicity, and inflammatory cascade activation (68). However, hyperactivated or unregulated microglia can cause neurotoxicity, which is an important source of pro-inflammatory factors and oxidative stress inducers, such as TNFs, nitric oxide, ILs, and other neurotoxic substances. Microglia have similar properties to peripheral macrophages, and activated microglia include inflammatory microglia (M1 type) and anti-inflammatory microglia (M2 type). M1-type activated microglia play a neurotoxic role by secreting reactive oxygen species and inflammatory cytokines. M2-type activated microglia produce anti-inflammatory cytokines and neurotrophic factors, which exert an anti-inflammatory and neuroprotective effect (69).

Our understanding of the role of microglia in SCI remains preliminary. Often, microglia responses are confused with macrophage responses. When the abundance of microglia reduces after SCI, it disrupts glial scar formation, increases parenchymal immune infiltration, decreases neuronal and oligodendrocyte survival, and inhibits locomotor function recovery (70). Meanwhile, the pharmacological depletion of microglial cells reduces neuroinflammation in the brain and spinal cord after injury, and improves cognitive recovery, depression-like behavior, and motor function (71). The transplantation of microglia in the area with SCI in an acute period increases tissue sparing, but does not lead to functional recovery (72).

#### 3.3.2 Immune chemicals released by microglia

Cytokines and chemokines released by activated microglia can induce the secretion of inflammatory factors and cytotoxic substances from leukocytes and macrophages, mediating neuroinflammation and neurotoxicity, and causing BBB disintegration and glial cell death. The sustained activation of M1-phenotype microglia causes excessive inflammatory factor and neurotoxic molecule secretion, leading to the death of normal cells and further damaging the tissue. Under treatment with lipopolysaccharides or interferon-gamma (IFN-γ), M1 microglia secrete pro-inflammatory cytokines, such as IL-1β, IL-2, IL-6, and TNF-α. Induced by IL-4 or IL-13, M2-type microglia secrete anti-inflammatory cytokines, such as IL-10, arginase 1, and TGF-β, which play a neuroprotective role and contribute to neuronal regeneration (70). Cytokines and chemokines released by activated microglia can induce leukocytes and macrophages to release inflammatory factors and cytotoxic substances, mediating neuroinflammation and neurotoxicity. This leads to the destruction of the BBB, and causes glial cell death (67). Recently, cytokines released by activated microglia after CNS injury occurrence have been found to influence the neurotoxic or neuroprotective effects of astrocytes (39). Aberrant growth factors such as IGF-1 are produced by the elimination of microglia. This leads to an increase in neuronal and oligodendrocyte death and a decline in locomotor performance (71).

### 3.4 Macrophages

#### 3.4.1 The roles of M1 and M2 macrophages in spinal cord injury pathophysiology

Macrophages are one of the primary cells that infiltrate to the lesion after SCI. These cells produce and secrete inflammatory factors, thereby aggravating secondary injury (73). Based on the molecular phenotype and function, macrophages can be classified into two major subsets: classically activated pro-inflammatory (M1) cells and alternatively activated anti-inflammatory (M2) cells (74). M1 macrophages exhibit proteolytic activity, expressing TNF-α and inducible nitric oxide synthase (iNOS). M2 macrophages express unique molecular markers, possess immunomodulatory, phagocytic, and remodeling properties, and mediate tissue repair (73, 75). There is evidence that both M1 and M2 macrophages infiltrate lesions in SCI. The spinal cord environment favors the polarization of macrophages toward a predominantly M1 cytotoxic macrophage phenotype (76). Evidence from previous studies has shown that M1 macrophages are neurotoxic, whereas M2 macrophages promote axonal regeneration (77–80). Therefore, increasing the M2 cell population at the site of injury may be a promising strategy to repair tissue damage post SCI (81). In the subsequent stage, macrophages reduce edema and induce the formation of a cavity by removing dead cells and myelin debris (80).

Macrophages play pivotal roles in recognizing and degrading cellular and tissue debris. Furthermore, macrophages remove the debris and components inhibiting myelin function. Therefore, macrophages can promote remyelination and axonal regeneration. However, foam macrophages appear when excess lipids accumulate in cells; this results in dysregulated lipid metabolism. This can lead to further neurological decline (73). The metabolic fitness of macrophages is conducive to secondary damage, and strategies that promote oxidative phosphorylation may help mitigate the negative effects of macrophages in nerve injury (82). M2 macrophage-derived exosomes loaded with berberine could be used to treat SCI *via* the inhibition of M1 inflammatory activation and anti-apoptosis function (83).

#### 3.4.2 Immune chemicals released by macrophages

Interferon-c (IFN-c) and prototypical T-helper type 1 cytokine can activate macrophages to produce cytotoxic mediators (such as reactive oxygen and nitrogen species) and pro-inflammatory cytokines [iNOS, IFN-c, TNF-α, C–C motif chemokine ligand 5 (CCL5), IL-6, IL-12, IL-6], and enhance their ability to kill pathogens present within cells. By contrast, the IL-13, IL-4, and T-helper type 1 cytokines inhibit macrophages from producing pro-inflammatory cytokines (15). Among these immunomodulatory chemicals, TNF-α is a protein with multiple functions. Targeting this cytokine could facilitate better SCI repair owing to its widespread inflammatory nature. TNF-α is a cytokine associated with acute and chronic inflammation. Predominant and prolonged TNF-α expression is counterproductive to post-SCI recovery (84). iNOS catalyzes the synthesis of nitric oxide, which participates in the apoptosis of neurons in the spinal cord (85). iNOS may exert a beneficial effect in the acute phase but plays a detrimental role in the chronic stage (86). TGF-β, also called human cytokine synthesis inhibitory factor, participates in cell differentiation and proliferation, which is mainly activate through type 1 and type 2 serine/threonine kinase receptors (87). In addition, TGF-β induces cellular apoptosis *via* activation of the SMAD pathway. IL-10 prevents the expression of IFN-c and TNF-α by keeping a check on CD8^+^ T cells (88) and inhibits MHC-II expression on the surface of monocytes/macrophages, which are involved in the presentation of antigens to T cells (89).

### 3.5 T and B cells

#### 3.5.1 The roles of T and B cells in spinal cord injury pathophysiology

T and B cells are two fundamental cells in adaptive immunity (90). SCI can activate T and/or B cells to induce autoimmune responses in the nervous system and maintain their activation for a long duration (91). There are different classification methods for T cells, including surface markers, transcriptional regulators, effector molecules, and functions. Based on the surface marker, T cells can be classified as CD4^+^ T cells and CD8^+^ T cells. The subpopulations of CD4^+^ T cells include helper T cells (Th1, Th2, and Th17) and regulatory T cells (Tregs) (90). T-cell infiltration in injured spinal cord tissues has been consistently reported in various SCI animal models (92). Jennifer et al. reported that a few CD8^+^ T cells were detected in human postmortem injured spinal cords, in both blood vessels and extravascular spaces, from hours to months after injury. The authors did not detect any CD20^+^ B cells in injured spinal cords (93). These findings were consistent with a recent study showing that CD8^+^ T cells are the major cell type among the few cells detected at the site of injury. Interestingly, CD138^+^/IgG^+^ plasma cells were found in a subpopulation of SCI, which serve as a reserve cell source in humoral immunity (82). Whether T cells cause secondary degeneration or mediate wound repair after SCI remains highly controversial. CD8^+^ T cell-derived perforin aggravates secondary SCI by damaging the BSCB (94). Chronic SCI impairs primary CD8^+^ T-cells’ antiviral immunity, but does not affect the generation or function of memory CD8^+^ T cells (95). Sirtuin 4 suppresses the anti-neuroinflammatory activity of infiltrating regulatory T cells at the SCI site (96). Programmed cell death protein 1 was shown to be essential for maintaining the anti-inflammatory function of infiltrating regulatory T cells in a murine SCI model (97). γδ T cells serve as an early source of IFN-γ to aggravate lesions in SCI. γδ T-cell recruitment to the SCI site promotes inflammatory responses and exacerbates neurological impairment (98). CCL2/CCR2 signaling is a vital mechanism underlying the recruitment of γδ T cells to the SCI site (99). SCI can trigger a demyelinating response, producing myelin basic proteins that activate T cells. Many cell adhesion molecules appear on the surface of activated T cells, which is useful for adhesion to vascular endothelial cells and to enable T cells to enter the CNS, inhibit axonal death, and promote neuroprotection (100).

The role and effect of B cells contributing to neurological changes after SCI occurrence has not been clarified. B cells can generate pathogenic antibodies that inhibit local lesion repair (90), leading to delayed recovery of neurological function. Precision therapeutic strategies that target B cells or block the effects of pathogenic antibodies have been proven to be effective (101). During the acute stage of injury, B cells in the bone marrow and spleen are substantial reduced. This is likely to be attributable to the reduction of B cell production, as the number and frequency of B cell progenitors in the bone marrow and periphery decrease at 8 days following SCI (15). The function of activated B cells that produce pro-inflammatory factors and maintain autoreactive T cells improves after B-cell knockout, but B cells also promote repair after SCI through their immunoregulatory Breg phenotype. Breg cells control antigen-specific T-cell autoimmune responses by producing IL-10 (91).

#### 3.5.2 Role of immune factors released by T and B cells in spinal cord injury pathophysiology

Lymphocytes are the only cell type that can specifically recognize the antigens and initiating the adaptive immune response (15). However, even though mechanisms by which these autoreactive T cells are eliminated or inactivated are existent, these are inadequate and autoreactive phenomenon is observed after SCI. T and B cells are responsible for the induction of autoimmunity in individuals. After induction of protective autoreactivity, a process characterized with immune response modulation by neural-derived peptides, the presence of T cells with a Th2 phenotype in the lesion site favors functional recovery (102, 103). This is because T cells have the ability to synthesize nerve growth factor (NGF), brain-derived neurotrophic factor (BDNF), and diverse neurotrophies (104, 105). CD4^+^ and CD8^+^ T cells in tandem with natural killer (NK) cells are the major sources of IFN-γ. There is a greater axial migration of T-cells in SCI mice, which is associated with an increase in macrophage/microglial activation and divergent expression patterns of growth factors and immune regulatory molecules (106). B cells benefit SCI repair by transforming to Breg phenotype, which regulate T cells’ autoimmune responses by controlling IL-10 production (107).

## 4 Potential pharmaceutical immunotherapeutic drugs for spinal cord injury

### 4.1 Methylprednisolone

Methylprednisolone is a classic drug recommended by SCI standard guidelines (108). A series of clinical trials over recent decades has demonstrated motor score improvements in patients with SCI treated with methylprednisolone. In addition, the 24-hour National Acute Spinal Cord Injury Study II (NASCIS II) dosing protocol has been shown to be relatively safe in SCI patients (109). Methylprednisolone treatment could inhibit the expression of TNF-α and the activity of NF-κB in SCI rats and promote the restoration of neurological function. Methylprednisolone can maintain biological activity in injured tissues and significantly reduce the inflammatory response and protein expression caused by secondary injury, thereby reducing edema (110, 111). Gao et al. have shown that methylprednisolone combined with amniotic mesenchymal stem cells suppressed MPO activity and cell apoptosis, reduced the expression of pro-inflammatory factors such as TNF-α, IL-1β, and IL-6, and increased the level of IL-10 (112). Methylprednisolone has been used in the treatment of SCI for more than 20 years, but owing to the major problems with respect to the time of treatment and dose, the appropriateness of its use has been debated. Therefore, for treating SCI using methylprednisolone, it is necessary to strictly control the dosage and time of administration to relieve pain. Methylprednisolone exhibits substantial immunosuppressive activity by increasing immune cell apoptosis and suppressing inflammatory responses (113). Furthermore, methylprednisolone can inhibit lipid peroxidation and protect oligodendrocytes from apoptosis-mediated cellular death after SCI. Methylprednisolone significantly attenuates the release of various inflammatory cytokines as well as the activation of T lymphocytes in symptomatic patients with multiple sclerosis (114). More importantly, methylprednisolone has been found to diminish the IL-12 levels in the CNS (115), indicating its competency for reducing autoreactive T-helper 1 lymphocyte reactions.

### 4.2 Minocycline

Minocycline is a lipophilic derivative of tetracycline that can penetrate the BBB (116). Casha et al. reported a single-center, double-blind, randomized clinical trial of minocycline administration after SCI. A total of 12 months after minocycline treatment, the motor function recovery of patients with SCI improved (117). Kobayashi et al. confirmed in an SCI mouse model that minocycline inhibited the expression of M1-type microglia surface markers and the generation of inflammatory cell factors *in vivo* and *in vitro*, but did not affect the expression of M2-type microglial surface markers *in vivo*. This is the first study to demonstrate the selectivity of minocycline in microglia subsets (118). The study showed that minocycline selectively inhibits the activation of microglia into pro-inflammatory states, which provides a basis for understanding the pathological changes that accompany many diseases involving microglia activation (118). Minocycline can achieve neuroprotection and functional recovery by eliminating secondary SCI injury (119).

### 4.3 Statins

Statins are inhibitors of 3-hydroxy-3-methylglutaryl-CoA (HMG-CoA) reductase. They can competitively combine with HMG-CoA reductase and inhibit cholesterol biosynthesis (120). Chung et al. reported that hyperlipidemia adversely affects the recovery of neurological function in patients with SCI. Therefore, theoretically, statins may reverse neurological disability in patients with SCI (121). However, in 2017, the only clinical trial investigating the efficacy of atorvastatin in patients with acute SCI found no significant improvement at the 3- and 6-month follow-up in patients receiving the drug (122). Nevertheless, various basic research studies have demonstrated the neuroprotective role of statins and elucidated the underlying molecular mechanisms. In 2014, Nacar et al. showed that the intraperitoneal administration of atorvastatin improved the recovery of locomotor activity in a rat SCI model constructed by single-level laminectomy at T10. Furthermore, atorvastatin reduced the levels of IL-1β, IL-6, and lipid peroxide in SCI rats. This indicates that atorvastatin affects SCI by modulating inflammatory cytokines (11). A single dose of atorvastatin used immediately after SCI inhibits inflammation and apoptosis and stimulates axon outgrowth, which may be important for functional outcomes improvement (123). Mirzaie et al. showed that lovastatin remarkably improved the functional outcomes of SCI treatment by reducing inflammation-induced tissue destruction, neuronal apoptosis, and demyelination post SCI. These findings indicated the neuroprotective role of lovastatin in SCI (124). Lovastatin could inactivate toll-like receptor 4 (TLR4) in microglia by targeting its co-receptor myeloid differentiation protein 2 (MD2) and alleviate neuropathic pain (125). However, there was a lack of robust neurological benefits with simvastatin or atorvastatin treatment after acute thoracic contusion SCI (126). More clinical research needs to be performed on this topic.

### 4.4 Human immunoglobulins

Immunoglobulins are a class of globulins that act as antibodies or have a chemical structure similar to that of antibodies. They are the primary reactive components in humoral immune response (127). Intravenous immunoglobulin (IVIG) has been used to treat various neurological diseases, but the evidence on its effects in SCI treatment is limited. In 2015, in a pioneer clinical trial named STRIVE (standard therapy for the treatment of transverse myelitis in adults and children), researchers attempted to investigate the efficacy of IVIG in SCI caused by infection. However, the study ended before completion because of the challenges associated with conducting a trial on a rare disease with a short enrolment window (128, 129). In 2018, another research team demonstrated that the intrathecal injection of neurite growth-promoting anti-Nogo-A antibodies contributed to neurological recovery in acute SCI (130). The effects of human immunoglobulin G in the recovery of tissue injury and neurological outcome after traumatic cervical SCI are with the involvement of the neurovascular unit (131). The delayed injection of high-dose human immunoglobulin G improves the outcome of traumatic cervical SCI *via* attenuating neuroinflammation and protecting the BSCB integrity (132).

### 4.5 Other potential immunomodulatory drugs under basic research

In the past few years, several basic research studies have been conducted on immunomodulatory options, which may be the focus of therapeutic strategies for SCI in the future. Neuroimmunophilin ligands are a class of compounds that have great potential in the treatment of neurological diseases, which easily penetrate the BBB. Several drugs targeting neuroimmunophilins, such as cyclosporin A, FK-506, and FK1706, have shown significant positive effects after SCI (133–135). Following SCI, the blockade of IL-6 signaling can facilitate the production of alternatively activated macrophages, and thereby alter the inflammatory response after SCI and promote spinal cord regeneration with functional recovery (136). Immunomodulatory therapy of SCI with ChABC yielded significant neuroprotective and potential neuroregenerative effects (137). Histone deacetylase (HDAC) is widely accepted as an enzyme that can eliminate acetyl groups from lysine residues localized on histone proteins and suppress transcription and gene expression (138). HDAC inhibitors, CI-994 inhibitors (139), RGFP966 (140), and valproic acid (141) were shown to exert significant neuroprotective effects in SCI. Finally, drugs targeting T and B cells like FTY720 also show beneficial effects in SCI (142). Furthermore, advanced therapies based on stem cells (112, 143) and new biomaterials that improve the tissue microenvironment are also promising direction (144, 145). **Table 1** shows recent findings on inflammation and immunotherapy with potential application in SCI. This may provide further guidance in both the research and the treatment for SCI.

**Table 1:** Summary of key clinical trials on immunotherapy after spinal cord injury (SCI).

Clinical trials							
Treatment	Country	Phase of study	Design of study	Study completion	Trialregistration	Conclusion	Ref
Methylprednisolone							
NASCIS I	USA	N/A	Multicenter, double-blinded RCT	1984	N/A	No difference in neurological recovery was observed between standard-dose and high-dose groups	(146)
NASCIS II	USA	N/A	Multicenter, double-blinded RCT	1990	N/A	Methylprednisolone improves neurological recovery, but naloxone does not improve neurological recovery after acute SCI	(147)
NASCIS III	USA	N/A	Double-blind, randomized	1997	N/A	Motor function improvement in patients who received a 48-h methylprednisolone infusion starting at 3–8 h after injury	(148)
Minocycline	Canada	Phase III	Placebo controlled, randomized	Be halted*	NCT01828203	Improvement of motor function	(117)
Statin	Iran	N/A	Placebo controlled, randomized	2015	2014032113947**	Improvement of sensory and motor function	(122)
Human immunoglobin							
Anti-Nogo-A antibody	Switzerland	Phase II	Multicenter, RCT	Dec. 2023	NCT03935321	Promoting axonal regeneration and neurobehavioral recovery	(130)
Anti-RGMa-antibody	Japan	Phase II	Double-blinded RCT	Mar. 2023	NCT04295538	Enhanced recovery of manual dexterity	N/A
Basic research							
Model/Species	Drug	Mode of administration	Dose	Interval	Outcome	Mechanism	Ref
SCI/Rat	Cyclosporin A		10 mg/kg	Every 12 h for 3 days	Improved neurological function	Inhibiting immune responses and mitochondrial permeability transition through both calcineurin-dependent and calcineurin-independent mechanisms	(135)
SCI/Rat	FK-506	Subcutaneous	2 mg/kg	Daily for 11 weeks	Improved neurological function	Stimulating retrograde-labeled neurons in the red nucleus	(133)
SCI/Rat	FK1706	Subcutaneous	2 mg/kg	Daily for 11 weeks	Improved neurological function	Stimulating retrograde-labeled neurons in the red nucleus	(133)
SCI/Mice	IL-6 inhibitor	IP	50 μg/g	Once	Improved neurological function recovery	Promoting the formation of alternatively activated M2 macrophages	(136)
SCI/Rat	FTY720	With polycaprolactone membrane	0.5 or 3 mg	Weekly for 4 weeks	Less cavitation volume and neuron loss, improved recovery of motor function	Decreasing S1P1 expression and glial scarring	(142)
SCI/Rat	ChABC	Injury site	6 μl (10 U/ml)	Alternate days for 10 days	Improved functional recovery of locomotor and proprioceptive behavior	Promoting regeneration of both ascending sensory projections and descending corticospinal tract axons	(46)
SCI/Mouse	CI-994 inhibitor	IP	1, 10, or 30 mg/kg	Daily for 14 days	Improved recovery of neurological function	Suppressing neutrophil accumulation, inflammatory cytokine expressions, and neuronal loss	(139)
SCI/Mouse	RGFP966	IP	10 mg/kg	2, 24, and 48 h after injury	Improvement of motor function	Dampened inflammatory cytokines	(140)
SCI/Rat	Valproic acid	IP	400 mg/kg	Daily for 7 days	Improvement of locomotor function	Reduction in secondary damage	(141)

*The study was discontinued owing to poor patient enrolment.

**This registration number is from the Iranian Registration of Clinical Trial Center (IRCT).

SCI, spinal cord injury; RCT, randomized controlled trial; ChABC, chondroitinase ABC; IP, intraperitoneal.

## 5 Limitations and perspectives

In the past decades, there have been significant advances in elucidating post-SCI immune responses and their relationships with other molecular mechanisms such as inflammation, axon regeneration, and scar formation. Nevertheless, there remain several limitations in research published to date: (i) there is a lack of a clear temporal–spatial profile of immune cells and immunological factors post SCI, (ii) there is a lack of substantial evidence on the safety of new immunomodulatory drugs, (iii) studies on novel drug carriers, such as nanomaterials, are promising but still at the nascent stage, and (iv) there is lack of sensitive and specific detection methods for clinical use for monitoring the progression of SCI. Therefore, more basic and clinical research should be undertaken, with a focus on the following: (i) immune cells and their roles at different time points and positions in the tissue microenvironment post SCI should be characterized more precisely, (ii) the safety of emerging drugs should be investigated thoroughly to ensure that physiological functions are not affected with their use, (iii) the application of new biomaterials to transport immunomodulatory drugs should be explored and characterized, and (iv) detection methods with high sensitivity and specificity should be developed to reflect comprehensive immune components after SCI. Collectively, as various experimental therapies are being developed or have been explored in clinical trials, promising therapies for SCI are expected.

## Author contributions

YG and XJ designed and drafted the manuscript. HT, LJ, GZ, and ZP revised the manuscript. All the authors finalized the paper and provided suggestions to improve it. All authors contributed to the article and approved the submitted version.
